# Detection of breastmilk antibodies targeting SARS-CoV-2 nucleocapsid, spike and receptor-binding-domain antigens

**DOI:** 10.1080/22221751.2020.1858699

**Published:** 2020-12-27

**Authors:** D. M. Favara, M. L. Ceron-Gutierrez, G. W. Carnell, J. L. Heeney, P. Corrie, R. Doffinger

**Affiliations:** a Department of Oncology, Addenbrooke’s Hospital, Cambridge University Hospitals NHS Foundation Trust, Cambridge, UK; b Department of Oncology, The Queen Elizabeth Hospital, The Queen Elizabeth Hospital King’s Lynn NHS Foundation Trust, Kings Lynn, UK; c Department of Oncology, University of Cambridge, Cambridge, UK; d Department of Immunology, Addenbrooke’s Hospital, Cambridge University Hospitals NHS Foundation Trust, Cambridge, UK; e Laboratory of Viral Zoonotics, Department of Veterinary Medicine, University of Cambridge, Cambridge, UK

**Keywords:** SARS-CoV-2, COVID-19, antibody, IgG, IgA, breastmilk

## Abstract

A 40-year-old female was found to have strongly neutralizing SARS-CoV-2 breastmilk IgA and IgG antibodies reactive against multiple SARS-CoV-2 antigens at 2.5 months after documented infection with SARS-CoV-2. At 6.5 months following the infection, she remained positive for breastmilk and serum SARS-CoV-2 neutralizing antibodies. Holder breast milk pasteurization did not diminish SARS-CoV-2 antibody titres but it reduced its neutralizing capacity, while serum heat inactivation had no negative effect on SARS-CoV-2 serum antibody levels and neutralizing capacity. Current data on SARS-CoV-2 and breastmilk are reviewed.

A 40-year-old breastfeeding female developed classical symptoms of SARS-CoV-2 infection (fever; fatigue, anosmia) in April 2020, with infection confirmed via a positive nasopharyngeal swab PCR testing. At the time of her illness, she temporarily stopped breastfeeding and isolated from her 16-month child and her husband. Her husband subsequently developed similar symptoms and was diagnosed following a positive SARS-CoV-2 nasopharyngeal swab PCR, while her infant displayed no signs of illness. Approximately 2 weeks later, she had a negative SARS-COV-2 nasopharyngeal swab PCR test and resumed breastfeeding. Her child continued to display no signs of infection.

In June 2020, she participated in a COVID-19 serology study (The CSOS Study [1]) and was noted to have high levels of anti-SARS-CoV-2 IgG antibodies using two different antibody assays: a rapid lateral flow point-of-care (POC) IgG test and a Luminex test using multiple SARS-CoV-2 antigens to detect reactive IgG and IgA. She also underwent repeat nasopharyngeal swab SARS-COV-2 PCR testing which was negative. The Luminex test showed that her high-level serum IgG response was directed against the SARS-CoV-2 nucleocapsid (N)-antigen, spike (S)-antigen, and receptor binding domain (RBD)-antigen, consistent with a vigorous humoral response to previous symptomatic and PCR-positive SARS-CoV-2 infection.

Persistently, high-level anti-SARS-CoV-2 IgG antibodies (targeting both N and S-antigens) were again detected in her blood 4 weeks later in July 2020 (2.5 months after infection). Nasopharyngeal swab PCR testing was again negative. At this time-point, she requested that her breastmilk also be tested for SARS-CoV-2 antibodies. Results confirmed both IgA and IgG antibodies against SARS-CoV-2 in her breastmilk, targeting the SARS-COV-2 N-antigen, S-antigen and RBD-antigen (Figure 1(a)). The IgA component predominated in her breastmilk **(**Figure 1(a)**)** while the IgG component predominated in her serum (Figure 1(b)). Breastmilk from three SARS-CoV-2 antibody-negative breastfeeding mothers was used as a negative control. Sera from 25 pre-pandemic healthy controls (collected between 2003 and 2008) were used as a negative control, while a reference SARS-CoV-2 antibody-positive serum (UK National Institute for Biological Standards and Control (NIBSC)) was used as a positive control. Higher anti-SARS-CoV-2 IgG antibody levels were detected in her serum than her breastmilk. Conversely, we found higher levels of anti-SARS-CoV2 antibodies in the breast milk, when compared to serum. This is in line with secretory IgA being the most abundant Ig in human milk, representing more than 90% of the total breast milk Ig [2].

**Figure 1.**
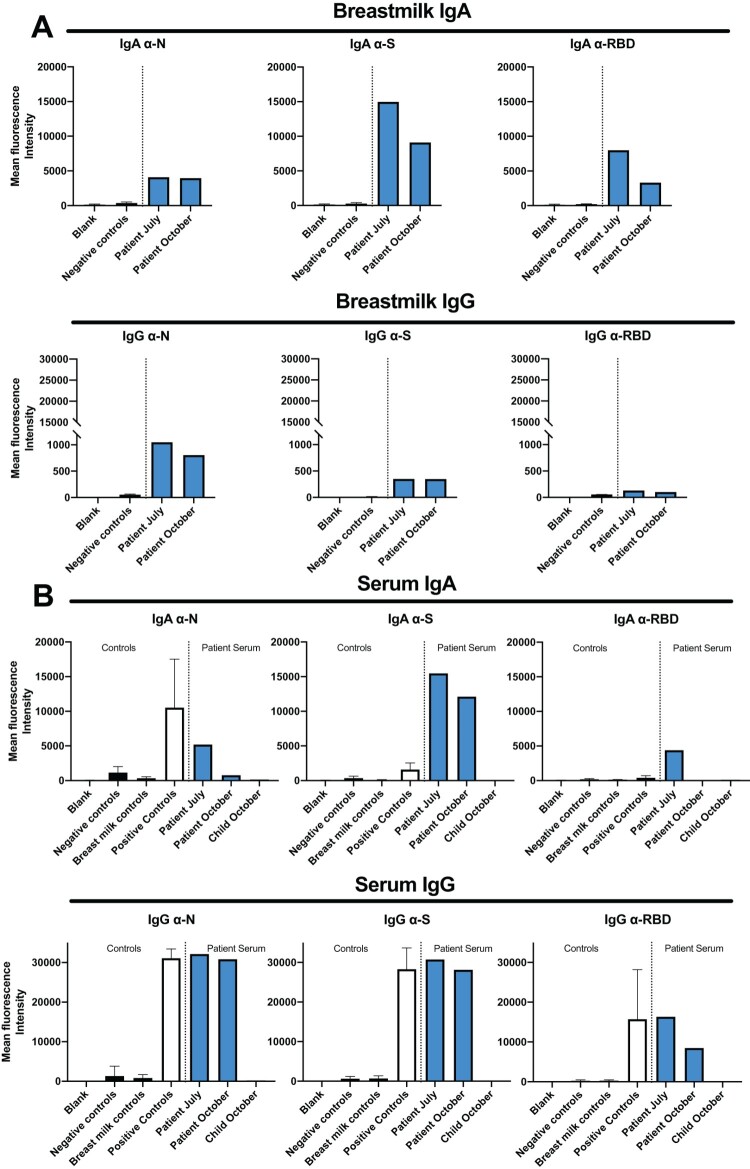
Anti-SARS-CoV-2 IgA and IgG antibodies in breast milk and serum. (A) Breastmilk SARS-CoV-2 IgA and IgG results to N, S and RBD at 2.5 and 6.5 months after SARS-CoV-2 infection. Black columns: negative controls (breastmilk from three SARS-CoV-2 antibody-negative breastfeeding mothers). Blue columns: index case’s breastmilk sampled during July and October 2020. All breastmilk samples were diluted 1:100. (B) Serum SARS-CoV-2 IgA and IgG results. Black columns: negative controls from 25 pre-pandemic control sera taken between 2003 and 2008 or breastmilk from three SARS-CoV-2 antibody-negative breastfeeding mothers. White columns: positive control (NIBSC SARS-CoV-2 serum positive controls). Blue columns: index case’s sera sampled during July and October 2020 and serum collected from infant in October 2020. All serum samples diluted 1:100.

In October 2020 (6.5 months after SARS-CoV-2 infection), the index case’s breastmilk and serum were retested, showing persistently positive SARS-CoV-2 antibody levels (Figure 1(a,b)). Her child’s serum was found to be SARS-CoV-2 antibody-negative for both IgG and IgA targeting SARS-CoV-2 N-antigen, S-antigen and RBD-antigen (Figure 1(b)). As Holder pasteurization of breastmilk (heating breastmilk to 62.5°C for 30 min) has been shown to successfully eradicate replication-competent SARS-CoV-2 virus experimentally added to breastmilk (as evidenced by negative viral cultures and PCR tests) [3], we tested whether the index case’s serum and breastmilk SARS-CoV-2 antibody levels would be affected by 30 min of heating. Results showed no change in detection levels between room temperature versus heated samples for both serum and breastmilk samples collected in July and October 2020 (Supplementary Figure 2A,B). In these experiments, serum was heated to 56°C for 30 min (higher temperatures caused immunoglobulin aggregation), whilst breastmilk was heated to 62.5°C for 30 min. Next, neutralization assays were performed using a SARS-CoV-2 S-antigen-expressing pseudovirus. Results showed that the patient’s SARS-CoV-2 antibodies in both serum and breastmilk (July and October 2020) were strongly neutralizing (Supplementary Figure 2C-D; IC50 levels: Supplementary Figure 2E). Heating had little effect on the neutralizing capability of serum SARS-CoV-2 antibodies (Supplementary Figure 2C). In contrast, heating lowered the neutralizing capability of breastmilk SARS-CoV-2 antibodies (Supplementary Figure 2D). For the neutralization assays, sera and breastmilk from three breastfeeding SARS-CoV-2 seronegative females were used as controls. To date, the index case has had no further SARS-CoV-2 symptoms or illness and continues to breast-feed her infant who remains healthy.

## Discussion

A small number of reports have shown that antibodies against SARS-CoV-2 can be detected in the breastmilk of breastfeeding women who have recovered from documented SARS-CoV-2 infection [4-6]. The largest of these reports investigated 37 breastfeeding women with recent documented SARS-CoV-2 infection finding that all (100%) had detectable SARS-CoV-2 IgA and IgG [7], while another investigating 15 breastfeeding women found that 12/15 (80%) had detectable IgA and IgG reactive to the SARS-COV-2 RBD-antigen [4]. Two other reports describe two individual patients: a pregnant woman with active SARS-CoV-2 infection who delivered an infant who had detectable IgA and IgG in her breastmilk [5], and a breastfeeding woman found to have SARS-CoV-2 IgG and IgM in her breastmilk (IgA was not tested) [6]. To the best of our knowledge, our case is the first to show that breastmilk SARS-CoV-2 antibody levels do not substantially decrease following Holder pasteurization and still retain neutralizing potential.

Breastmilk is rich in antibodies (predominantly isotype IgA) as well as a range of additional antimicrobial factors providing long-lasting passive immunity to neonates and infants [8,9]. IgA secreted in breastmilk coats the infant’s gut once swallowed, neutralizing any pathogenic viruses and bacteria and protecting the infant from infection until it is able to secrete its own intestinal IgA (usually after 4 weeks of age) [10]. IgG and IgM antibody isotypes are also present in breastmilk although at lower concentrations. This fits with our case who had a predominantly IgA antibody response in her breastmilk, whilst having a predominantly IgG response in her serum with low-level IgG detectable in her breastmilk.

Interestingly, antibodies are relatively resistant to proteolytic digestion in the gut compared to other proteins [9] as evidenced by studies showing 19% of ingested IgG and IgM being detectable in the terminal adult ileum [11] compared to 7% of ingested milk proteins [12]. This also applies to infants where 52% of the IgA concentration of ingested breastmilk has been detected in gut samples collected 2 h after ingestion [8]. Furthermore, even partially-degraded IgA, IgG and IgM antibodies remain functional and bind antigen if their Fab portion remains intact, leading to target degradation and excretion from the gut [9]. This suggests that any neutralizing SARS-COV-2 targeting antibodies present in breastmilk may have a protective effect for the recipient infant, provided that the infant has not already mounted their own immune response to the infection. Furthermore, gut Fc receptors may transport antibody from the gut into the vascular circulation, further aiding systemic defences.

At present, the World Health Organization (WHO) recommends that breastfeeding women with suspected or confirmed SARS-COV-2 infection be encouraged to continue breastfeeding as the benefits outweigh the risks [13]. Holder pasteurization has been shown to eradicate viruses from breastmilk, including SARS-CoV-2 virus [3,14]. Our results show that whilst it does not affect antibody levels in either serum or breastmilk, it decreased the neutralizing capacity of breastmilk SARS-CoV-2 antibodies whilst having no negative effect on serum SARS-CoV-2 neutralization. The reason for this is unclear and could be related to the effect of heating on other breastmilk components leading to aggregation and trapping of a proportion of breast milk antibodies. Despite the decrease in breastmilk antibody neutralizing capacity following Holder pasteurization, neutralizing activity was still present which suggests that heat-inactivated SARS-CoV-2 neutralizing antibody-positive breast milk may be of protective benefit to breastfeeding children of mother’s who have active and severe SARS-CoV-2 infection.

There has been speculation regarding the transmission of SARS-CoV-2 virus through breastmilk since the first report of detectable SARS-CoV-2 RNA in breastmilk [15]. However, a recent case series of 18 breastfeeding women with documented SARS-CoV-2 infection was unable to detect replication-competent virus in the breastmilk of the 1 participant who had detectable SARS-CoV-2 RNA in her breastmilk [3]. She subsequently became RNA negative later suggesting that her infection had been cleared. This picture will become clearer as more cases are documented.

## Supplementary Material

Supplemental Material

supplementary_data-clean.docx
