# Supplementary material for: Detection of breastmilk antibodies targeting SARS-CoV-2 nucleocapsid, spike and receptor-binding-domain antigens
Source: Emerg Microbes Infect. 2020 Dec 27;9(1):2728–31. doi: 10.1080/22221751.2020.1858699 (PMC7782901; doi:10.1080/22221751.2020.1858699)
Supplement: Supplemental Material [file TEMI_A_1858699_SM6695.pdf]

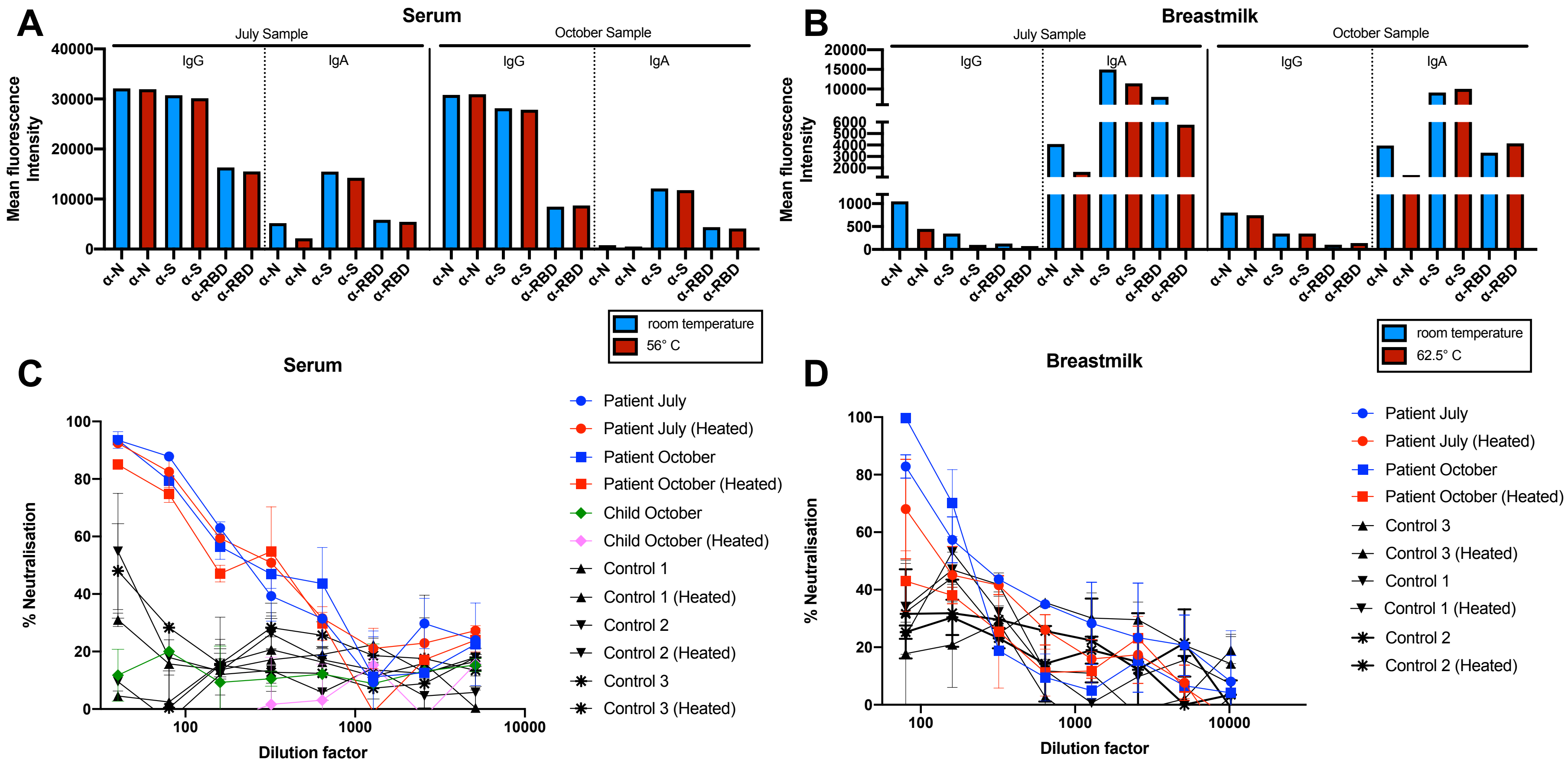

E

Serum	IC50	Breast Milk	IC50
Patient July	310.7	Patient July	322.3
Patient July (Heated)	358.3	Patient July (Heated)	174.7
Patient October	311.8	Patient October	209.9
Patient October (Heated)	232.8	Patient October (Heated)	54.36
