# Supplementary material for: Detection of breastmilk antibodies targeting SARS-CoV-2 nucleocapsid, spike and receptor-binding-domain antigens
Source: Emerg Microbes Infect. 2020 Dec 27;9(1):2728–31. doi: 10.1080/22221751.2020.1858699 (PMC7782901; doi:10.1080/22221751.2020.1858699)
Supplement: supplementary_data-clean.docx [file TEMI_A_1858699_SM1744.docx]

**Supplementary Data**

**Supplementary Figure 2.** **Thermostable neutralising SARS-CoV-2 antibodies present in both breastmilk and serum 2.5 and 6.5 months after SARS-CoV-2 infection.** Serum **(A)** and breastmilk **(B)** SARS-CoV-2 (N, S, RBD) IgG and IgA detection at room temperature and following heating. Blue columns: antibodies levels at room temperature; red columns: antibody levels following 30 minutes of heating at 56°C for serum samples and 62.5°C for breastmilk samples. Serum **(C)** and breastmilk **(D)** SARS-CoV-2 antibody neutralising assays. Index case samples collected during July and October 2020: blue (room temperature), red (following 30 minutes of heating at 56°C for serum samples and 62.5°C for breastmilk samples). Three breastfeeding SARS-CoV-2 negative (PCR and antibody) women provided control samples. (**E**) Neutralisation IC50 values for the index case’s SARS-CoV-2 positive serum and breastmilk.

**Methods:**

## **Luminex antibody detection**

The serum negative control comprised of pre-pandemic control sera from 25 healthy control taken between 2003-2008. The serum positive control comprised UK National Institute for Biological Standards and Control (NIBSC) SARS-CoV-2 positive controls (NIBSC 20/130). The breast milk negative control comprised of breastmilk from three SARS-CoV-2 antibody-negative breastfeeding mothers. All samples were diluted 1:100.

Negative antibody status for both negative control populations was assessed using a multiplexed bead array (Luminex) test using multiple SARS-CoV-2 antigens Nucleocapsid protein (N), Trimeric spike protein (S) and receptor binding domain protein (RBD) as targets to detect reactive IgG and IgA.

N, S and RBD proteins were covalently coupled to distinctive carboxylated bead sets (Luminex) to form a three-plex assay. Beads were first activated with 1-ethyl-3-(3-dimethylaminopropyl)carbodiimide hydrochloride (Thermo Fisher Scientific) in the presence of *N*-hydroxysuccinimide (Thermo Fisher Scientific), according to the manufacturer’s instructions, to form amine-reactive intermediates. The activated bead sets were incubated with the corresponding proteins at a concentration of 50 μg ml^−1^ in the reaction mixture for 3 h at room temperature on a rotator. Beads were washed and stored in a blocking buffer (10 mM PBS, 1% BSA, 0.05% NaN_3_).

The S, N and RBD coupled bead sets were incubated with SARS-CoV-2 patient sera or breast milk at a 1/100 dilution for 1 h in 96-well filter plates (MultiScreenHTS; Millipore) at room temperature in the dark on a horizontal shaker. Fluids were aspirated with a vacuum manifold and beads were washed three times with 10 mM PBS/0.05% Tween 20. Beads were incubated for 30 min with a PE-labeled anti-human IgG-Fc antibody (Leinco/Biotrend), washed as described above, and resuspended in 100 μl PBS/Tween. They were then analysed on a Luminex analyser (Luminex/R&D Systems) using Exponent Software V31. Specific binding was reported as mean fluorescent intensity (MFI).

**Pasteurisation of serum and breastmilk**

Holder Pasteurisation of the breast milk was performed in a waterbath at 62.5°C for 30 minutes. Heat treatment of the serum was done instead at 56 C (as it would coagulate at 62.5°C).

## **Lentiviral pseudotype production**

Luciferase lentiviral pseudotypes bearing the SARS-CoV-2 glycoprotein were produced as described previously with several modifications[1,2]. Sub-confluent HEK293T/17 cells in 6-well format were transiently transfected with lentiviral packaging plasmids p8.91[3,4], pCSFLW[5] and an expression plasmid bearing the full length SARS-CoV-2 spike gene with no modifications, using the FuGENE-HD (Promega) transfection reagent at a 1µg:3µl DNA to volume ratio. Lentiviral pseudotypes were titrated on HEK293T/17 cells transiently transfected with pCAGGS-hACE-2[6] and pCAGGS-TMPRSS2[7] 48h before assay, and results read in relative luminescence units (RLU) 48 after transduction of target cells.

## **Pseudotype based microneutralisation assay (pMN)**

Pseudotype based microneutralisation assay was performed as described previously[8]. Serial dilutions of serum or breast milk were incubated with SARS-CoV-2 lentiviral pseudotypes for 1h at 37°C, 5% CO2 in 96-well white cell culture plates. HEK293T/17 cells (obtained from Dr Edward Wright (University of Sussex, Brighton, UK) transfected 48h previously with pCAGGS_hACE-2 and pCAGGS-TMPRSS2 were added to the 96-well plate, at a density to reach 1.5x10^4^ cells per well. Plates were then incubated for 48h at 37°C, 5% CO_2_ in a humidified incubator. Bright-Glo (Promega) was then added to each well and luminescence read after a five-minute incubation period. Experimental data points were normalised to 100% and 0% neutralisation controls and compared to the NIBSC 20/130 research reagent (National Institute for Biological Standards and Control, UK).

**Method References:**

1. Carnell GW, Grehan K, Ferrara F, et al. An Optimized Method for the Production Using PEI, Titration and Neutralization of SARS-CoV Spike Luciferase Pseudotypes. Bio-protocol. 2017 2017/08/20;7(16):e2514.

2. Temperton NJ, Hoschler K, Major D, et al. A sensitive retroviral pseudotype assay for influenza H5N1-neutralizing antibodies. Influenza Other Respir Viruses. 2007 May;1(3):105-12.

3. Zufferey R, Nagy D, Mandel RJ, et al. Multiply attenuated lentiviral vector achieves efficient gene delivery in vivo. Nature biotechnology. 1997 Sep;15(9):871-5.

4. Naldini L, Blomer U, Gage FH, et al. Efficient transfer, integration, and sustained long-term expression of the transgene in adult rat brains injected with a lentiviral vector. Proceedings of the National Academy of Sciences of the United States of America. 1996 Oct 15;93(21):11382-8.

5. Zufferey R, Dull T, Mandel RJ, et al. Self-inactivating lentivirus vector for safe and efficient in vivo gene delivery. Journal of virology. 1998 Dec;72(12):9873-80.

6. Hoffmann M, Kleine-Weber H, Schroeder S, et al. SARS-CoV-2 Cell Entry Depends on ACE2 and TMPRSS2 and Is Blocked by a Clinically Proven Protease Inhibitor. Cell. 2020 Apr 16;181(2):271-280 e8.

7. Bertram S, Glowacka I, Blazejewska P, et al. TMPRSS2 and TMPRSS4 facilitate trypsin-independent spread of influenza virus in Caco-2 cells. Journal of virology. 2010 Oct;84(19):10016-25.

8. Carnell GW, Ferrara F, Grehan K, et al. Pseudotype-based neutralization assays for influenza: a systematic analysis. Frontiers in immunology. 2015 2015/04/29;6(MAR):161.
